# Human quadrupedalism is not an epiphenomenon caused by neurodevelopmental malformation and ataxia

**DOI:** 10.3389/fneur.2012.00154

**Published:** 2012-10-25

**Authors:** Sibel Karaca, Meliha Tan, Uner Tan

**Affiliations:** ^1^Department of Neurology, Adana Teaching and Medical Research Center, Başkent University Medical SchoolAdana, Turkey; ^2^Department of Physiology, Çukurova University, Medical SchoolAdana, Turkey

**Keywords:** quadrupedalism, Uner Tan syndrome, neural networks, self-organization, evolution

## Abstract

Two cases with quadrupedal locomotion (QL) were presented. In both cases, cognitive and psychiatric functions were normal and, no neurological deficits were observed, except for a sequel paralysis of left leg in Case 2. It was suggested that human QL (1) should not be considered as an epiphenomenon caused by neurodevelopmental malformation and ataxia, but (2) may be considered as a re-emergence of the ancestral diagonal QL, and (3) it may spontaneously emerge in humans with entirely normal brains, by taking advantage of neural networks such as central pattern generators that have been preserved for about 400 million years.

## Introduction

Habitual human quadrupedalism was first described in 2005, as the most impressive symptom of Uner Tan syndrome (see for a review Tan et al., 2012). Namely, patients with no childhood hypotonia first crawled on their hands and knees like normal children, but adopted quadrupedal locomotion (QL) instead of bipedal locomotion during their locomotor development. They could stand up, but lost their balance while making a step to initiate upright walking, despite having strong muscles in their arms and legs, and no hypotonia.

All of the patients exhibited dysarthric speech with a limited vocabulary or just a few sounds, but they could understand simple requests. All patients were mentally impaired, and in most cases their brain MRI's and PET scans showed a cerebellovermian hypoplasia with mild simplification in the cortical gyri.

In discussions of the origins of the habitual QL observed in Uner Tan syndrome, it was argued that this quadrupedalism might be an epiphenomenon caused by neurodevelopmental malformation and severe truncal ataxia (Herz et al., 2008). The present work will show that this argument may be untenable, presenting two individuals with QL who do not exhibit ataxia, and who have entirely normal brain images and cognitive functions.

## Case histories

Two case histories of individuals who adopted QL are presented in this work. Neurological examinations were performed by two of the authors (Sibel Karaca and Meliha Tan). The mini-mental state examination test (MMSE) was used to assess their cognitive integrity. Written informed consent was obtained from the parents of Case 1 and, himself of Case 2 before presentation of this study and associated videos.

**Case 1** was a 12-year-old boy, who had been delivered by caesarian section after a normal gestational period. After a period of crawling on hands and knees, at two years of age he started to adopt well-coordinated QL for fast locomotion. He was able to walk and run upright without any sign of imbalance but he preferred running on all fours for fast locomotion, such as during playing with his father or hurrying to the WC on waking at night.

He used diagonal sequence when walking or running on all fours, with the right foot touching the ground followed by the left hand, and the left foot touching the ground followed by the right hand (Shapiro and Raichien, 2005), a gait favored by our early ancestors (Reilly et al., 2006). **Video 1** shows the bipedal and QL of Case 1 when he was 4-years-old.

There were no other instances of QL among his close family or relatives. The marriage between his parents was not consanguineous.

**Figure 1:**
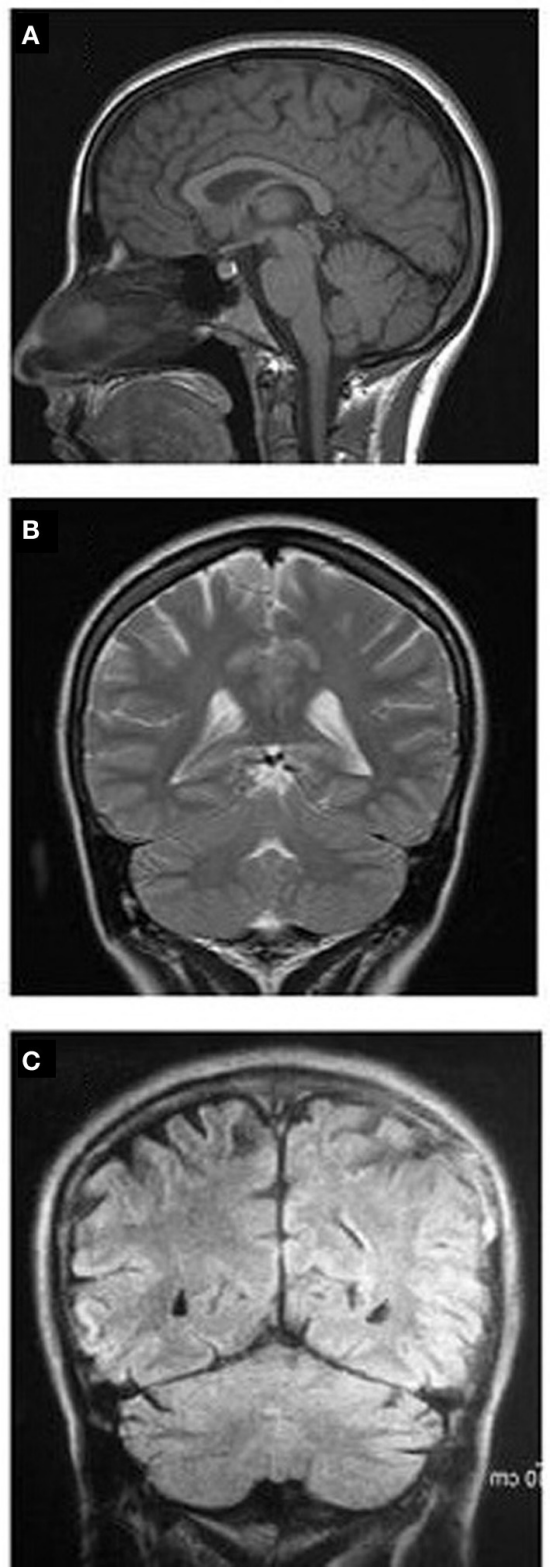
**MRI images showing sagittal (A) and coronal (B) sections of case 1 with facultative QL; (C) coronal MRI of case 2 with a paralyzed leg.** Notice the entirely normal brain structures in both cases.

The neurologic examination of Case 1 showed normal findings in motor, sensory (light touch, vibration and position), and cerebellar testing's. There was no past history of psychiatric symptoms and the results of a mental status examination (with a score of 30/30 in MMSE) did not reveal any mood or psychiatric disorder or other Axis I disorder according to Diagnostic and Statistical manual of Mental Disorders, 4th edition (DSM-IV). He spoke fluently with a large vocabulary. His measurements of height, weight, and head size were within normal ranges. His MRI scans (Figures 1A,B) were also in normal limits.

**Case 2** was a 28-year-old man who lived in Mugla in western Turkey and who habitually walked on all fours. This man had a paralyzed left leg due to infantile poliomyelitis, similar to an earlier case discovered in a small village near Adana in southern Turkey (Tan, 2007). There was no intra-familial marriage between his parents, and no other case of QL among his immediate or extended family.

His parents reported that he became paralyzed in his left leg in the course of an illness with high fever, at the age of three months. Like Case 1, this individual had an almost-normal crawling period (because of the sequel of paralyzed left leg), but he did not try to walk upright. He was given help to walk upright such as crutches and assistance to walk from his father, and he was also offered a wheelchair. He refused all assistance, preferring always to walk on all fours. All of his movements were well-coordinated.

The preferred method of locomotion of Case 2 was habitual diagonal-sequence wrist-walking, but he could use only three extremities because of his paralyzed leg. His locomotion is shown in **Video 2**.

Case 2's neurologic examination was consistent with residual-paralysis stage of poliomyelitis including deformities of left hip, knee, and foot; disuse atrophy of muscles and shortening of the left leg. The left leg tonus was flaccid and no sensory deficit was defined. Other three extremities and cranial nerves were absolutely normal in neurological examination. His memory functions were also normal as Case 1 with 30/30 score in MMSE. Mental status examination of Case 2 revealed that, there wasn't any mood or psychiatric disorder or other Axis I disorder according to DSM-IV. He spoke two languages (Turkish and English) fluently with a large vocabulary. Although, he walks on all fours, he was employed as a tourist guide in summer times. His brain MRI scan was normal (Figure 1C).

## Discussion

In this work we have presented two cases of habitual quadrupedalism: Case 1 exhibiting a facultative QL for fast locomotion only, and Case 2 exhibiting habitual quadrupedal wrist walking.

There are millions of individuals with a paralyzed leg, but none reported to exhibit QL. So walking on all fours in such individuals is an extremely rare phenomenon. Case 1, an entirely normal child preferring QL for rapid locomotion, is also extremely rare.

In Uner Tan syndrome, the obligate QL was associated with some genetic mutations and cerebellovermial hypoplasia, and was seen as an adaptive self-organizing response to limited balance (Ozcelik et al., 2008; Tan et al., 2012). On the other hand, the present work showed that human QL may spontaneously occur in humans with an unimpaired brain, probably using the ancestral locomotor networks for the diagonal sequence QL, preserved for about the last 400 million years (Shapiro and Raichien, 2005; Reilly et al., 2006).

Contrary to the argument considering human QL as an adaptive epiphenomenon resulting from developmental malformations and ataxia (Herz et al., 2008), human QL in the individuals with entirely normal brains is consistent with the dramatic role of a self-organizing brain during locomotor development (Thelen et al., 1987; see also Tan et al., 2012). Accordingly, the coordination between the arms and legs during upright walking of human beings reflects the ancestral coupled oscillators, also seen during QL (Wannier et al., 2001). So the ancestral human QL can be considered as a reflection of the activity of the ancestral neural networks, and not as a simple adaptive response to limited balance resulting from developmental malformations and ataxia, as argued by Herz et al. (2008).

## Supplementary material

The Supplementary Material for this article can be found online at: http://www.frontiersin.org/Movement_Disorders/10.3389/fneur.2012.00154/abstract

**Video S1. A four-year-old child with facultative diagonal sequence QL, with the right foot touching the ground followed by the left hand, and the left foot touching the ground followed by the right hand.** He runs on all fours for fast locomotion, and walks upright for everyday actions.

**Video S2. A man with a paralyzed leg habitually wrist-walking on all fours.** He was able to walk and run easily and at speed with no sign of imbalance, and could negotiate steps.

### Conflict of interest statement

The authors declare that the research was conducted in the absence of any commercial or financial relationships that could be construed as a potential conflict of interest.
